# Development and Content Validation of a Self-Assessment Checklist for Electronic Medical Record Use Certification in Korea: Convergent Mixed Methods Study

**DOI:** 10.2196/95500

**Published:** 2026-11-04

**Authors:** Joohyun Chung, Ye-Eun Park, Yoomi Kim, Jae-Ho Lee, Yura Lee

**Affiliations:** 1 College of Nursing, University of Massachusetts Amherst, Amherst, MA, United States; 2 Department of Information Medicine, University of Ulsan College of Medicine, Asan Medical Center, 88 Olympic-ro 43-gilSeoul, 05505, Republic of Korea, 82 2-3010-1498, 82 2-3010-2531; 3 Electronic Medical Records System Certification Criteria Development Department, Korea Health Information Service, Seoul, Republic of Korea; 4 Department of Emergency Medicine, University of Ulsan College of Medicine, Asan Medical Center, Seoul, Republic of Korea

**Keywords:** electronic medical record, certification, patient safety, clinical workflow, content validity, self-assessment, clinical decision support

## Abstract

**Background:**

Electronic medical record (EMR) certification programs have traditionally focused on product-level requirements, such as interoperability, security, and core functionality. However, growing evidence suggests that many patient safety risks associated with EMRs arise after implementation and are closely related to system configuration, workflow integration, and real-world use. Despite the presence of a national EMR product certification system in Korea, a systematic evaluation of how certified systems are implemented and used in clinical practice is still lacking.

**Objective:**

This study aimed to develop and validate a self-assessment checklist for EMR use certification, focused on patient safety-critical functions, and to evaluate its content validity and expert-assessed practical applicability across health care institutions of different sizes.

**Methods:**

A convergent mixed methods design was used. A self-assessment checklist comprising 10 patient safety–related EMR functions was developed by adapting the US Office of the National Coordinator for Health Information Technology self-assessment templates to the Korean EMR certification context. Content validity was quantitatively assessed using the Content Validity Index across 4 dimensions (relevance, usefulness, clarity, and importance) by a panel of 24 experts with experience in EMR implementation and certification. Qualitative feedback was collected through open-ended survey questions to examine feasibility, anticipated challenges, and differentiation between product and use certification across tertiary referral hospitals, general hospitals, and clinics.

**Results:**

The overall Scale-Level Content Validity Index was 0.921, indicating strong content validity for the proposed checklist. Although most items demonstrated high agreement, lower ratings were observed for certain functions, particularly in clinic-level settings, reflecting structural and resource-related constraints rather than limited recognition of patient safety priorities. Qualitative findings highlighted challenges related to staffing, system governance, and the feasibility of assembling multidisciplinary assessment teams in smaller institutions.

**Conclusions:**

The self-assessment checklist demonstrated high content validity and was perceived as relevant for evaluating patient safety–critical aspects of EMR use. However, expert-assessed feasibility varied across health care institution types, particularly in clinic-level settings. Further empirical testing is needed to assess the checklist’s reliability, usability, and applicability in real-world certification processes.

## Introduction

The adoption of electronic medical record (EMR) systems has accelerated worldwide, highlighting the need for mechanisms to ensure the safety, quality, and interoperability of digital health infrastructures. International organizations such as the World Health Organization (WHO) emphasize the importance of robust digital health strategies, governance, trust, and reliable information exchange across health care settings [1,2]. Importantly, digital transformation in health care must be accompanied by safeguards that protect patients from technology-related errors and unintended consequences [3-5]. As EMRs become integral to clinical decision-making, medication management, communication, and care coordination, ensuring their safe use has emerged as a critical patient safety priority [5,6].

Many countries have introduced EMR certification programs that primarily evaluate product-level requirements, including interoperability, privacy and security, and core functionality. In the United States, the Office of the National Coordinator for Health Information Technology (ONC) operates a national health IT certification program that defines technical and functional standards for certified health IT [7]. However, technical conformance alone does not ensure the safe and effective use of EMR systems in clinical practice. Prior studies, sociotechnical frameworks, and recent reviews have emphasized that health IT–related safety risks often emerge after implementation through system configuration, workflow integration, alert design, user interaction, and EMR usability problems [8-11]. Technology-induced errors may contribute to medication errors, delayed diagnoses, communication failures, documentation inaccuracies, and other patient safety events. Research has shown that these risks often arise from mismatches between EMR design and clinical workflows, inadequate governance processes, and unintended consequences of system customization rather than from software malfunctions alone [12-15]. Complementary frameworks such as the Healthcare Information and Management Systems Society’s electronic medical record adoption model further illustrate the importance of evaluating how EMR systems are implemented and used within clinical organizations [16].

Korea has established a national EMR product certification program and promotes the use of standard terminologies and interoperability guidelines [17]. However, systematic approaches for assessing how certified EMR systems are configured, used, and governed in Korean clinical practice remain limited, despite broader evidence that postimplementation health IT risks are shaped by sociotechnical factors such as configuration, usability, workflow integration, and governance [18-20]. Korea has experienced early and rapid adoption of EMR systems, particularly in tertiary and general hospitals; however, this rapid diffusion has been accompanied by concerns about patient safety, system usability, interoperability, and workflow integration in Korean EMR environments [21-27]. Given that patient safety outcomes depend not only on the technical capabilities of EMR systems but also on how these systems are implemented and used in routine care, there is a growing need for mechanisms that can identify potential safety vulnerabilities before they contribute to adverse events. Such mechanisms may support health care organizations in strengthening safe EMR practices, promoting organizational learning, and reducing technology-related patient harm [12,14,28].

Taken together, international experience suggests the need for a complementary use certification framework that evaluates the appropriateness, clarity, and feasibility of EMR functions in real clinical settings [29-31]. A use-focused certification approach may provide an additional layer of patient safety assurance by evaluating whether safety-critical EMR functions are appropriately configured and supported by organizational governance, interoperable information exchange, workflow-aligned implementation practices, and adequate user training and support [32,33]. Such an approach recognizes that patient safety risks may emerge not only from technical deficiencies but also from interactions among system usability, information quality, workflow integration, clinician cognitive workload, and organizational factors that influence the safe and effective use of EMRs in routine care [3,4,34]. To address this gap in the Korean context, this study aimed to develop a self-assessment checklist for EMR use certification based on Korea Health Information Service (KHIS)–designated patient safety–critical certification items. Specifically, the study aimed to evaluate the content validity of the checklist using expert ratings across relevance, usefulness, clarity, and importance and examine expert-perceived feasibility across tertiary referral hospitals, general hospitals, and clinics.

## Methods

### Study Design

This study used a convergent mixed methods design to develop and validate a self-assessment checklist for EMR use certification within the Korean context. Quantitative data were used to assess the content validity of selected certification items, whereas qualitative feedback was collected to examine clarity, applicability, and alignment with real-world clinical practice. In this study, “EMR” is used as the primary term to reflect the terminology of the Korean certification program; “EHR” (electronic health record) is retained only when referring to international frameworks or source materials that use the term “EHR.”

### Participants and Recruitment

A purposive sampling strategy was used to recruit experts with direct experience in EMR implementation, evaluation, or certification in Korea. Of the 31 individuals invited, 24 experts with a response rate of 77.4% participated. Participants were affiliated with professional organizations, including the Korean Medical Informatics Association, the Korean Hospital Information Association, the Korean Society for Patient Safety, and a national health IT usability and safety research group.

Eligibility criteria included substantial experience with EMR systems, certification processes, and professional roles such as health care IT specialists, clinicians (physicians, nurses, and pharmacists), policymakers, or representatives of relevant associations. Snowball sampling was also used to broaden disciplinary representation. The sample size was selected based on recommendations by Lynn, who suggested that 5 to 10 experts are adequate for content validity evaluation, and by Polit and Beck, who recommended a minimum item-level Content Validity Index (I-CVI) threshold of 0.78 when 6 or more experts are involved [35,36]. The experts independently rated each item across 4 dimensions using a 4-point scale. I-CVI and scale-level Content Validity Index (S-CVI/Ave) were subsequently calculated. All participants provided informed consent prior to participation.

### Development of the EMR Use Self-Assessment Checklist

The self-assessment checklist was developed based on 10 patient safety-critical functional items identified from the Korean EMR product certification standards established by KHIS. The KHIS EMR certification system is divided into 3 major categories (interoperability, safety and security, and functionality) and includes 28, 20, and 12 detailed certification items, respectively (Figure 1). Among these 60 certification items, 10 were designated by KHIS as patient safety-critical functional items with high relevance for field verification. These KHIS-designated items served as the basis for developing and validating the EMR use self-assessment checklist. Drug Allergy and Hypersensitivity (A009) and Safe Prescription of Medication Dosages (A020) were retained as separate KHIS-designated items because they represent distinct medication-safety functions within the Korean EMR certification framework. A009 focuses on recording and reviewing medication allergy and hypersensitivity information, whereas A020 focuses on safe medication dosage prescribing, including patient-specific dose calculation, linkage between calculated doses and prescription entry, and dosage-range review. The details of these 10 items are shown in Table 1.

**Figure 1.**
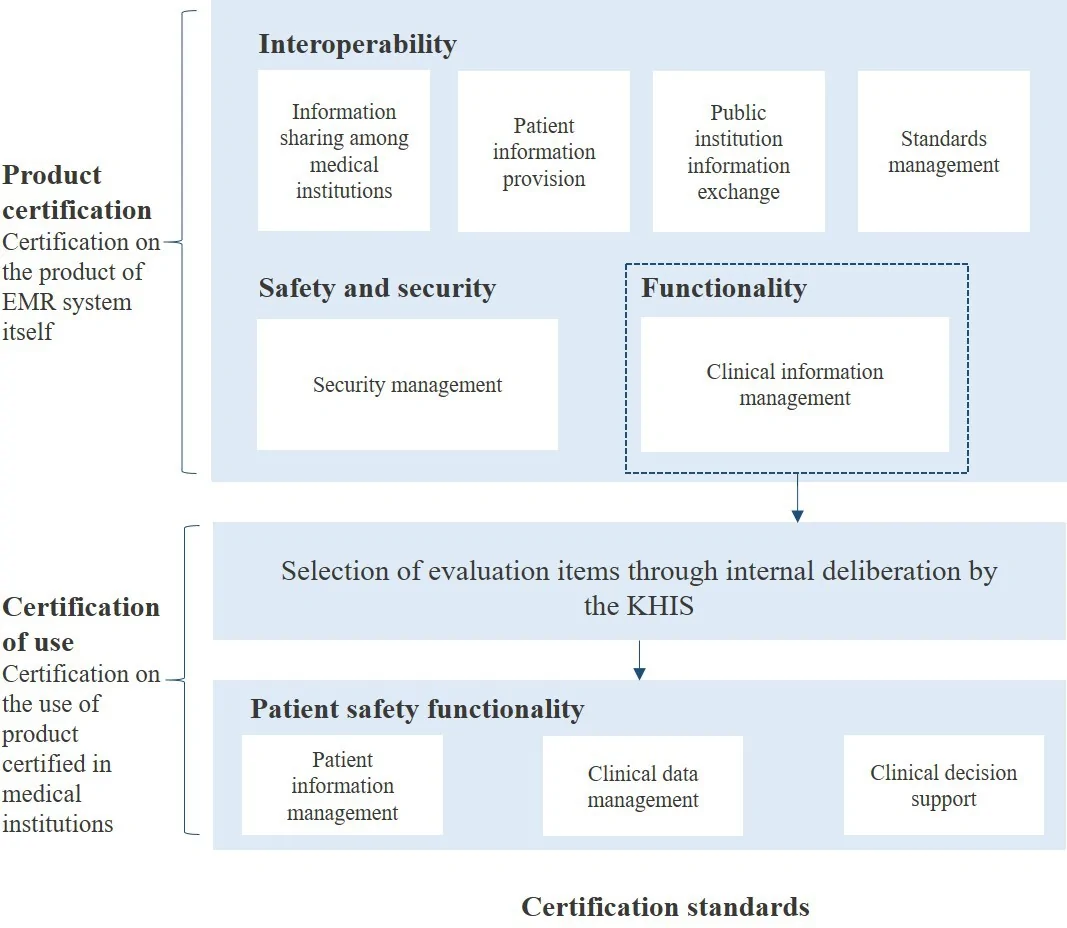
Overview of the electronic medical record (EMR) certification framework in Korea, including the structure of product and use certification and core evaluation domains (interoperability, safety and security, and functionality). KHIS: Korea Health Information Service.

**Table 1.** Ten certification items for the “certification of use” process.

Product certification code	Item	Definitions
A003	PI^a^	Ensures the system accurately identifies patients using multiple identifiers (eg, name, ID number, and date of birth)
A009	DAH^b^	The EMR^c^ must allow recording and reviewing of a patient’s allergies or hypersensitivities to medications
A017	DIA^d^	Provides alerts for potential adverse reactions when a drug is ordered that may interact with other medications or allergies
A018	MEP^e^	The system includes checks and balances (eg, autocomplete, dosage suggestions, and alerts) to reduce entry errors during medication ordering.
A019	IRAM^f^	Prevents or alerts users when a medication is ordered via an incorrect or dangerous route (eg, oral vs intravenous)
A020	SPMD^g^	Supports calculation and review of patient-specific medication dosages during prescribing to promote safe medication use
A021	BRMA^h^	Supports scanning barcodes or RFID^i^ tags on patient ID bands and medications during administration to verify correct patient, drug, and time
A022	MIP^j^	Provides clinicians and/or patients with detailed drug information (eg, dosage, side effects, and contraindications)
A024	CTRN^k^	Automatically alerts clinicians about life-threatening or time-sensitive test results (eg, abnormal laboratory values)
A025	APM^l^	Tracks antibiotic prescribing patterns, includes alerts for inappropriate use, and may support antimicrobial stewardship

a PI: Patient Identification.

b DAH: Drug Allergy and Hypersensitivity.

c EMR: electronic medical record.

d DIA: Drug Interaction and Allergy Alert.

e MEP: Medication Entry Error Prevention.

f IRAM: Inappropriate Route of Administration Management.

g SPMD: Safe Prescription of Medication Dosages.

h BRMA: Barcode or RFID Medication Administration.

i RFID: radio frequency identification.

j MIP: Medication Information Provision.

k CTRN: Critical Test Result Notification.

l APM: Antibiotic Prescription Management.

To enhance international alignment, the checklist was adapted from the US ONC self-assessment templates and modified to reflect Korean regulatory requirements, clinical workflows, and institutional contexts [7,17,29]. The adaptation process was conducted by 2 investigators and involved reviewing the ONC templates, mapping relevant elements to the Korean certification criteria, and revising wording and response fields for contextual relevance.

Disagreements regarding mapping or wording were resolved through research team discussion, and the revised checklist was reviewed by health care IT professionals for practical usability. The complete checklist, including item-specific purposes, assessment questions, implementation status options, improvement and management plan fields, and organizational responsibility checklists, is provided in Multimedia Appendix 1.

### Data Collection

Data were collected between December 2023 and March 2024 using a structured, web-based survey. Quantitative evaluation used the CVI framework [35], with participants rating each of the 10 certification items on a 4-point Likert scale across 4 dimensions: relevance, usefulness, clarity, and importance. All 24 experts were invited to rate each certification item separately for 3 health care institution types; I-CVI calculations were based on the number of valid ratings for each item-dimension-institution–type combination. Missing ratings were excluded only from the specific item-dimension-institution–type calculation in which they occurred. The expert validation survey was distinct from the clinical self-assessment checklist developed for future institutional use. In the expert survey, the unit of CVI evaluation was the certification item/domain, not the individual subquestions or organizational responsibility questions embedded in the checklist. These embedded questions were included as operational components to guide future institutional self-assessment of implementation status, governance, accountability, and readiness.

To assess pragmatic feasibility, participants also evaluated 3 implementation-related domains, referred to in this study as practical features: ease of completion, target respondent suitability, and content adequacy. These practical features were rated using 4-point scales, with higher scores indicating more favorable evaluations: ease of completion (1=“not easy” to 4=“very easy”), target respondent suitability (1=“insufficient” to 4=“fully sufficient”), and content adequacy (1=“negative” to 4=“positive”). As part of the summary evaluation, for each health care institution type, experts were asked to estimate the minimum number of participants or professional roles required to complete the 10 self-assessment forms.

Qualitative data for this study consisted of free-text responses to open-ended questions included in the expert validation survey. These questions addressed anticipated implementation challenges, differentiation from product certification, and applicability across clinical settings. These responses were distinct from the “improvement and management plan” fields embedded in the clinical self-assessment checklist, which were intended for future institutional completion during actual self-assessment and were not analyzed in this study.

### Data Analysis

Data were analyzed using SAS version 9.4 (SAS Institute Inc). I-CVI values were calculated at the certification item/domain level as the proportion of experts rating an item as 3 or 4. An I-CVI value ≥0.78 was considered acceptable [36]. I-CVI values were computed separately for each domain (clarity, importance, relevance, and usefulness) and for each health care institution type (tertiary referral hospitals, general hospitals, and clinics). S-CVI/Ave was computed as the average of the I-CVI values to assess overall content validity.

To adjust for chance agreement among experts, modified κ (κ*) coefficients were calculated for each item-dimension-institution–type combination. The probability of chance agreement was estimated based on the number of valid ratings and the observed level of agreement for each item-dimension-institution–type combination. κ* was computed as κ*=(I-CVI−Pc)/(1−Pc), where I-CVI indicates the observed proportion of agreement among experts and Pc indicates the probability of chance agreement [36-38]. Interpretation of κ* followed established criteria: ≥0.90, excellent agreement; 0.80 to 0.89, good agreement; 0.70 to 0.79, acceptable agreement; <0.70, poor agreement.

Descriptive statistics, including means and SDs, were calculated for practical feature ratings and summary evaluation items, including the estimated minimum number of participants or professional roles required to complete the self-assessment forms by health care institution type.

Qualitative responses from the open-ended survey questions were analyzed using qualitative content analysis with an inductive coding approach. Two investigators independently reviewed all responses, generated initial codes, and grouped similar codes into preliminary categories. Coding results were then compared to identify discrepancies in code assignment, category boundaries, and theme interpretation. Discrepancies were resolved through discussion and consensus between the 2 coders; when consensus could not be reached, a third investigator reviewed the relevant responses and adjudicated the final coding decision. The final themes were refined through iterative discussion among the research team to ensure that they reflected the participants’ comments and were aligned with the study objectives. Institution-type comparisons were conducted using repeated ratings from the same expert panel and were not based on mutually exclusive expert subgroups. Coding and category development were conducted using spreadsheet-based data matrices.

### Ethical Considerations

The study was reviewed by the Institutional Review Board of Asan Medical Center and was determined to be exempt from review (exemption number 2024‐1033). The study was conducted in accordance with relevant ethical guidelines. Participation was voluntary, informed consent was obtained from all participants prior to their involvement, and any information that could directly identify individual participants or their affiliated institutions was removed from the analytic dataset. Participants received an honorarium equivalent to approximately US $200 for their participation. No images or supplementary materials include identifiable individual participants, patients, users, or institutions.

## Results

### Participants

A total of 24 experts participated in the expert panel evaluation, including 4 patient safety specialists, 6 medical informatics professionals, 9 hospital information system practitioners, and 5 human factors experts (Table 2). Their academic backgrounds included IT (n=8), medicine (n=7), pharmacy (n=5), industrial engineering (n=2), and nursing (n=2). All participants had at least 10 years of professional or research experience in their respective fields and were recruited through recommendations from relevant academic societies or professional associations.

**Table 2.** Characteristics of the expert panel (N=24).

Characteristics	Values, n (%)
Sex	
Male	14 (58.3)
Female	10 (41.7)
Age (y)	
30‐39	2 (8.3)
40‐49	9 (37.5)
50‐59	12 (50)
60‐69	1 (4.2)
Professional specialty	
Hospital information system	9 (37.5)
Medical informatics	6 (25)
Human factors	5 (20.8)
Patient safety	4 (16.7)
Educational background	
IT	8 (33.3)
Medicine	7 (29.2)
Pharmacy	5 (20.8)
Industrial engineering	2 (8.3)
Nursing	2 (8.3)

### Quantitative Findings

I-CVI values ranged from 0.476 to 1.000 across item-dimension-institution–type combinations, with most combinations demonstrating acceptable to high content validity. Content validity was generally high for tertiary referral hospitals and general hospitals, whereas greater variability was observed for clinics. The mean I-CVI values for the 10 certification item domains ranged from 0.797 to 0.992 after averaging across evaluation dimensions and health care institution types (Figure 2). The overall S-CVI/Ave was 0.921, indicating strong content validity across the 10 certification items. The number of valid ratings ranged from 21 to 24 across item-dimension-institution–type combinations. The complete item-dimension-institution type values for I-CVI, probability of chance agreement (Pc), and modified κ* are provided in Multimedia Appendix 2. No item-level mean I-CVI fell at or below the predefined threshold of 0.78. However, 2 items (A021: Barcode or Radio Frequency Identification [RFID] Medication Administration and A025: Antibiotic Prescription Management) showed comparatively lower mean I-CVI values than the other certification item domains.

**Figure 2.**
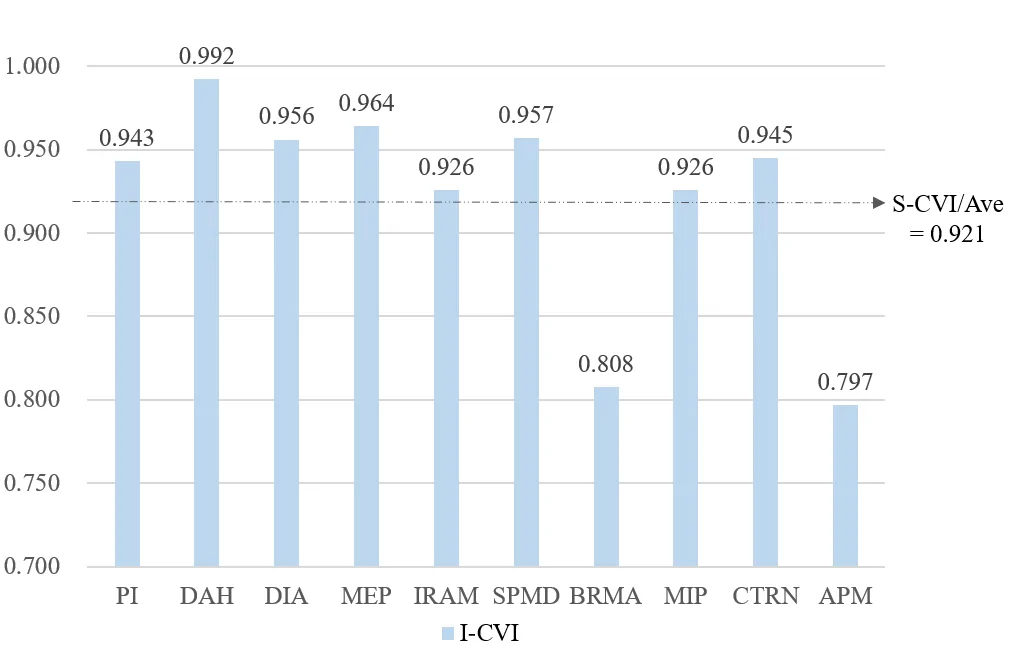
Item-level Content Validity Index (I-CVI) scores for the 10 electronic medical record use certification item domains. Bars represent item-level mean I-CVI values averaged across evaluation dimensions and health care institution types. The dashed horizontal line represents the S-CVI/Ave (0.921). The y-axis was scaled from 0.700 to 1.000, with the maximum set to 1.000, consistent with the possible upper bound of the Content Validity Index (CVI). APM: Antibiotic Prescription Management; BRMA: Barcode or RFID Medication Administration; CTRN: Critical Test Result Notification; DAH: Drug Allergy and Hypersensitivity; DIA: Drug Interaction and Allergy Alert; IRAM: Inappropriate Route of Administration Management; MEP: Medication Entry Error Prevention; MIP: Medication Information Provision; PI: Patient Identification; RFID: radio frequency identification; S-CVI/Ave: scale-level Content Validity Index; SPMD: Safe Prescription of Medication Dosages.

The average I-CVI score was highest for relevance (mean 0.942, SD 0.105), followed by clarity (mean 0.925, SD 0.095), importance (mean 0.925, SD 0.091), and usefulness (mean 0.894, SD 0.142), indicating relatively lower expert agreement regarding practical utility.

Modified κ* coefficients were computed for each combination of item, dimension, and institution type (tertiary referral hospitals, general hospitals, and clinics). Modified κ* coefficients were generally consistent with I-CVI estimates for high-agreement combinations, whereas larger differences were observed for several lower-scoring clinic-level combinations.

### Content Validity by Health Care Institution Type

Content validity differed by health care institution types. The mean I-CVI values were 0.974 (SD 0.032) for tertiary referral hospitals, 0.963 (SD 0.051) for general hospitals, and 0.827 (SD 0.140) for clinics. Clinics showed lower average I-CVI values than those of tertiary referral hospitals and general hospitals, with mean I-CVI values of 0.838 (SD 0.110) for clarity and 0.757 (SD 0.172) for usefulness (Figure 3). At the clinic level, several item-dimension combinations did not meet the predefined acceptability threshold of I-CVI≥0.78 [36]. Five of the 10 certification item domains had at least one clinic-level dimension below the threshold. A017 (Drug Interaction and Allergy Alert), A019 (Inappropriate Route of Administration Management), and A024 (Critical Test Result Notification) had below-threshold clinic-level usefulness scores of 0.762, 0.714, and 0.762, respectively. In addition, A021 (Barcode or RFID Medication Administration) scored below the threshold across all 4 dimensions: 0.667 for relevance, 0.476 for usefulness, 0.619 for clarity, and 0.667 for importance. A025 (Antibiotic Prescription Management) also scored below the threshold across all 4 dimensions: 0.524 for relevance, 0.476 for usefulness, 0.667 for clarity, and 0.667 for importance. These results show that clinic-level applicability concerns were most pronounced for A021 (Barcode or RFID Medication Administration) and A025 (Antibiotic Prescription Management), while usefulness concerns also extended to A017 (Drug Interaction and Allergy Alert), A019 (Inappropriate Route of Administration Management), and A024 (Critical Test Result Notification).

**Figure 3.**
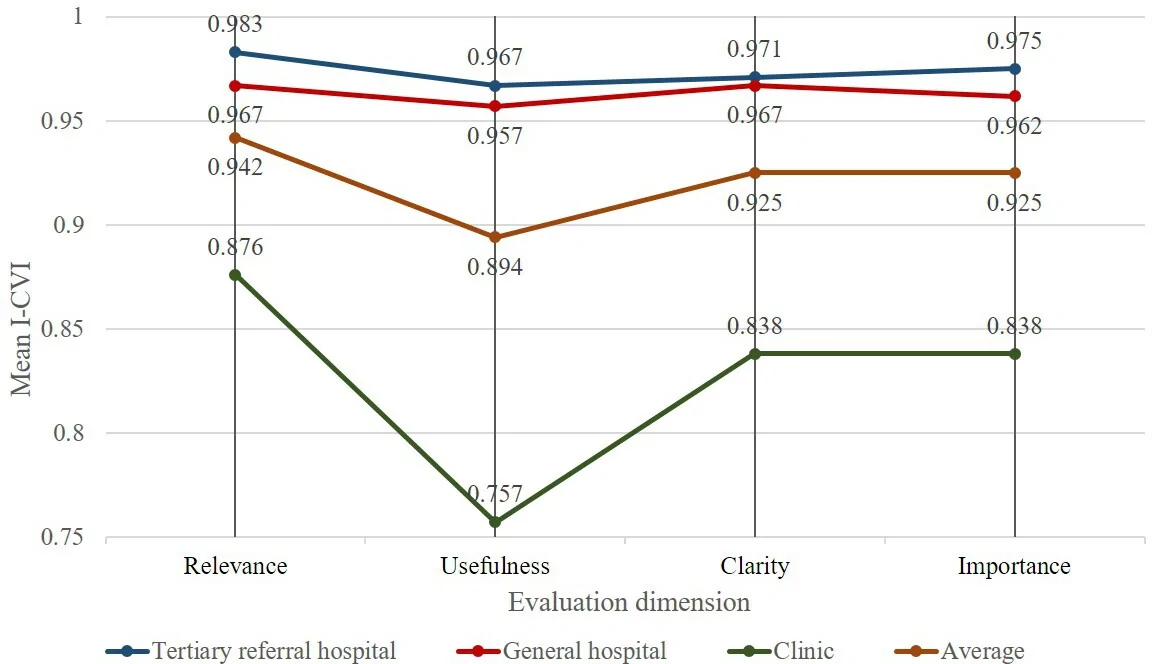
Item-level Content Validity Index (I-CVI) scores by health care institution type across 4 content validity dimensions: relevance, usefulness, clarity, and importance.

Regarding κ* coefficients, values for tertiary referral hospitals ranged from 0.917 to 1.000 across item-dimension combinations. κ* values ranged from 0.809 to 1.000 for general hospitals and from 0.370 to 1.000 for clinics. Across tertiary referral hospitals, general hospitals, and clinics, κ* values exceeded 0.900 for most item-dimension combinations across the 4 evaluation dimensions. The lowest modified κ values were observed for clinic-level A021 usefulness and A025 usefulness, both of which had a modified κ of 0.370. The probability of chance agreement ranged from approximately 0 to 0.168.

### Practical Feature Evaluation by Survey

In the expert-assessed practical feature evaluation, differences were observed across health care institution types. For target respondent suitability, the mean scores were 3.29 (SD 0.52) for tertiary referral hospitals, 3.04 (SD 0.52) for general hospitals, and 2.35 (SD 0.92) for clinics. For content adequacy, the corresponding scores were 2.75 (SD 0.56), 2.71 (SD 0.51), and 2.70 (SD 0.49). For ease of completion, the scores were 3.67 (SD 0.41), 3.44 (SD 0.57), and 2.92 (SD 0.91), respectively (Table 3).

**Table 3.** Mean (SD) scores of practical features across health care institution types.

Item and type of health care institution	Value, mean (SD)
Target respondent suitability	
Tertiary referral hospital	3.29 (0.52)
General hospital	3.04 (0.52)
Clinic	2.35 (0.92)
Content adequacy	
Tertiary referral hospital	2.75 (0.56)
General hospital	2.71 (0.51)
Clinic	2.70 (0.49)
Ease of completion	
Tertiary referral hospital	3.67 (0.41)
General hospital	3.44 (0.57)
Clinic	2.92 (0.91)

### Qualitative Findings

The thematic content analysis of open-ended expert feedback yielded 3 major themes: self-assessment as a preparatory mechanism for on-site evaluation, the conditional feasibility of self-assessment without on-site evaluation, and the need to distinguish between use certification and product certification. The results below summarize expert feedback corresponding to each theme.

#### Theme 1: Self-Assessment as a Preparatory Tool Prior to On-Site Evaluations

Most experts (n=16) expressed positive views regarding the potential utility of the self-assessment as a preparatory tool prior to on-site evaluations. They noted that, when used in this context, the checklist could serve as a valuable resource for preassessment. However, several limitations were identified. Specifically, concerns were raised about the usability of the self-assessment in smaller health care settings, particularly at clinics (eg, doctors’ offices). Experts suggested that separate self-assessments be developed for tertiary, secondary, and clinic-level institutions. Additionally, although the self-assessment is well-structured and easy to read, some content, particularly around medication safety, is repetitive and needs refinement. The clarification of vague terms, alignment with actual processes, and inclusion of implementation examples (eg, system screenshots or formats) were noted as potential considerations.

Experts emphasized that the pre–self-assessment should primarily serve as a tool to verify whether the EMR format is appropriate, whether it is being maintained and improved to support patient care and safety, and to confirm the presence of the necessary organizational infrastructure to sustain its use.

#### Theme 2: Conditional Feasibility of Self-Assessment Without On-Site Evaluation

Most experts (n=13) agreed that independent evaluations can be sufficiently used as an assessment tool, regardless of whether on-site evaluations are conducted. Self-assessments effectively verify compliance but have limited ability to drive necessary improvements for certification. They can complement but not replace on-site evaluations, which are essential owing to variations in question interpretation and response accuracy. Assessments conducted solely by clinicians (eg, physicians or nurses) are challenging, thereby emphasizing the need for clearer guidance and precise questions.

Experts identified several conditions that could enhance the feasibility of self-assessment, including clear identification of responsible personnel, explicit definitions within the evaluation criteria, and predefined thresholds for determining when follow-up on-site evaluation may be needed. Additionally, specifying department roles and encouraging detailed item-level participation were perceived as potentially enhancing efficiency. Including guidelines for question comprehension, requiring evidence submission (eg, screenshots), and verifying actual clinical use beyond the existence of a program were also identified as important considerations. For clinics without formal departmental structures, experts perceived that assigning responsible individuals and obtaining leadership confirmation may be particularly important for implementing the self-assessment process.

#### Theme 3: Need to Distinguish Use Certification From Product Certification

Several experts noted that the current self-checklist can be used for tertiary referral hospitals, but a separate use certification, distinct from product certification, may be unnecessary. Although improvements are possible, especially in assessing actual clinical use rather than just product features, the distinction between product and use certification remains unclear. Use certification should focus on verifying real-world applications, such as allergy registration and alert use, and incorporate data on actual system use rather than simply confirming feature availability. Additionally, evaluation criteria should be customized to reflect differences in workflows and medication management across different hospital types.

Although the evaluation forms are detailed, they can be difficult for smaller clinics and would benefit from simplification and clearer instructions. Providing additional support, such as clinic-specific guides, separate questionnaires for IT and clinical staff, and help desk services, would enhance the process. Experts suggested that evaluation teams may need to include both IT/system managers and clinical users to ensure that the assessment reflects both system functionality and clinical workflow. Additionally, focusing on usability, providing actionable feedback, and implementing effective scoring will improve adoption. Some assessment items may require adjustment to better reflect real-world conditions. Overall, the evaluation framework is solid but should be tailored to the institution’s size and role to increase clarity, usability, and effectiveness.

### Appraisee Composition

In the summary evaluation, experts estimated the minimum number of personnel or professional roles required to complete the 10 self-evaluation forms for each health care institution type. The estimated number was highest for tertiary referral hospitals (mean 3.42, SD 1.89), followed by general hospitals (mean 2.95, SD 1.10) and clinics (mean 2.20, SD 0.83). These values refer to expert-estimated minimum personnel requirements for checklist completion, not to actual study participants. However, responses also indicated that smaller medical institutions would be more difficult to evaluate, even when system administrators (IT personnel or related departments) and users (medical professionals) are involved, either alone or in collaboration.

### Expert Feedback on Certification Items Across Health Care Institution Types

First, experts expressed that tertiary care facilities need precise, standardized processes and advanced functionalities to ensure patient safety and clinical accuracy. Critical areas include rigorous patient identification protocols, allergy and drug interaction checks, and prevention of medication entry errors with a focus on look-alike/sound-alike drugs. Robust management of administration routes and integration of weight data for dose calculation are important but are challenged by workload and data accuracy. Barcode and RFID medication administration systems were recognized as key patient safety tools, but experts noted that their implementation requires substantial investment and institutional commitment. Antibiotic stewardship systems should prioritize alerts for restricted drugs, with flexible approval workflows acknowledging physician shortages. Although medication information provision and critical test-result notifications are valued, costs and clarity in guideline definitions remain concerns. Overall, tertiary referral hospitals require scalable, comprehensive solutions that balance safety, workflow efficiency, and resource constraints.

For general hospitals, experts noted challenges related to specialist availability and resource constraints. They require simplified, well-integrated tools that prioritize practical management over technical complexity. Patient identification and allergy documentation systems should ensure consistency but remain adaptable to fewer staff. Medication error prevention and administration route management need structured but feasible processes supported by pharmacy departments that may face staffing challenges. Barcode and RFID implementations are rare and often cost-prohibitive, necessitating external support and tailored investment plans. Antibiotic management must consider the absence of infectious disease specialists and focus on institution-specific restrictions. Medication information provision and test result notifications, although important, must be balanced against limited financial and operational capacity.

For clinics, experts noted that limited staffing and simpler prescribing workflows require highly practical and cost-effective solutions. Patient identification and allergy alert systems should be straightforward and easy to use, prioritizing usability over complexity. Medication entry error prevention and dose calculation features have limited utility because of the infrequent use of complex or high-risk drugs. Barcode and RFID systems are generally impractical due to cost, low drug complexity, and limited administration procedures. Antibiotic stewardship in clinics should focus on education and awareness, with flexible, low-burden monitoring rather than strict electronic controls. Medication information provision is largely the responsibility of pharmacies, and critical test-result notifications are rare and limited to basic omission checks. Overall, experts noted that clinic-level EMR use certification involves balancing safety features with simplicity, cost-efficiency, and workflow practicality.

### Integration of Quantitative and Qualitative Findings

To integrate the quantitative and qualitative strands, we developed a joint display that links expert feedback with corresponding quantitative results and mixed methods interpretations of checklist validity and feasibility (Table 4).

**Table 4.** Joint display integrating qualitative feedback and quantitative results on checklist validity and feasibility.

Qualitative explanation from expert feedback	Quantitative results	Integrated mixed methods interpretation
Cost burden, limited staffing, and infrequent medication administration procedures were identified as barriers to A021^a^ implementation in clinics.	A021 clinic I-CVI^b^ was below the threshold across all dimensions: relevance, 0.667; usefulness, 0.476; clarity, 0.619; importance, 0.667.	A021 may require clinic-specific adaptation because low ratings aligned with implementation and workflow constraints.
Limited infectious disease specialist support and need for low-burden approaches were identified as barriers to A025^c^ implementation in clinics.	A025 clinic I-CVI was below the threshold across all dimensions: relevance, 0.524; usefulness, 0.476; clarity, 0.667; importance, 0.667.	A025 may require lower-burden implementation models for clinics with limited specialist capacity.
Several items were described as clinically important but difficult to operationalize in smaller institutions.	“Usefulness” was the lowest-rated dimension: overall, 0.894; clinic-level, 0.757.	Experts distinguished clinical importance from practical implementability, particularly in clinics.
Documentation burden, evidence submission, and respondent identification were identified as barriers to completion.	“Ease of completion” was lower in clinics: 2.92 (SD 0.91) vs 3.67 (SD 0.41) in tertiary referral hospitals and 3.44 (SD 0.57) in general hospitals.	Completion burden was more pronounced in clinics than in larger institutions.

a A021 refers to the Barcode or RFID Medication Administration item.

b I-CVI: item-level Content Validity Index.

c A025 refers to the Antibiotic Prescription Management item.

## Discussion

### Principal Findings

This study identified high overall content validity for the proposed self-assessment checklist for EMR use, while also showing variation in expert-assessed usefulness and feasibility across health care institution types, particularly in clinical settings.

### Interpretation and Implications

The expert panel’s evaluation demonstrated high overall content validity, with an S-CVI/Ave of 0.921. This finding suggests that the selected KHIS-designated certification items were generally perceived as relevant, important, clear, and useful for evaluating patient safety-critical aspects of EMR use. Across the 4 evaluation dimensions, relevance showed the highest agreement, whereas usefulness was relatively lower, indicating that experts distinguished the clinical importance of the items from their feasibility in routine implementation.

The lower clinic-level ratings were not limited to A021 (Barcode or RFID Medication Administration) and A025 (Antibiotic Prescription Management). Although these 2 items fell below the predefined I-CVI threshold across all 4 evaluation dimensions, clinic-level usefulness scores were also below the threshold for A017 (Drug Interaction and Allergy Alert), A019 (Inappropriate Route of Administration Management), and A024 (Critical Test Result Notification). This pattern suggests that clinic-level concerns were concentrated not only in resource-intensive medication administration and antibiotic stewardship functions but also in the perceived usefulness of selected decision-support and notification functions. These findings strengthen the need for tiered, capacity-sensitive implementation guidance rather than a uniform certification approach across all health care institution types. Qualitative feedback suggested that these lower ratings may reflect concerns related to implementation cost, staffing, workflow structure, and limited specialist support. For example, these findings are consistent with qualitative feedback, indicating that medication safety functions may be viewed as clinically important but difficult to operationalize in smaller institutions.

The institution-type differences observed in this study support the need for context-sensitive interpretation of EMR use certification items. Tertiary referral hospitals and general hospitals showed higher average I-CVI values than clinics, whereas clinic-level ratings indicated lower perceived applicability and practical usefulness for selected functions and greater difficulty in identifying appropriate respondents or responsible personnel. These findings do not necessarily indicate that the underlying patient safety objectives are less important in clinics. Rather, they suggest that workflow fit, implementation requirements, organizational responsibility, and availability of resources may differ across institution types. These patterns suggest that a uniform self-assessment approach may not adequately reflect differences in organizational capacity, governance structures, and available technical support across health care settings.

These findings can be interpreted through sociotechnical perspectives on health IT safety, which emphasize that safety risks may emerge from interactions among technology, users, workflows, and organizational context rather than from software functionality alone [8-10,18,30]. The checklist items examined in this study—patient identification, medication safety, decision support, critical result communication, and antibiotic stewardship—therefore represent patient safety-critical areas in which both technical functions and real-world use conditions require attention [39-43].

Medication safety-related items accounted for several of the lower-rated certification items and item-level evaluations, highlighting the importance of context-sensitive interpretation. Closed-loop medication management, barcode or RFID-based medication administration, clinical decision support alerts, and critical result notification are widely recognized as important safety functions, but their effectiveness depends on workflow fit, alert configuration, documentation practices, escalation protocols, and organizational accountability [14,40,41,44]. Expert feedback in this study similarly indicated that these functions may be clinically important but difficult to implement uniformly across health care institution types, particularly in clinics with limited staffing, specialist support, or medication administration workflows.

Organizational capacity emerged as a critical determinant of expert-assessed feasibility. One possible explanation is that general hospitals and clinics may have fewer dedicated IT personnel, less formalized governance structures, and more limited capacity for continuous EMR optimization than tertiary referral hospitals. The consistently lower clinic-level ratings, particularly for identifying appropriate respondents or responsible personnel, may reflect these structural constraints rather than a lack of recognition of patient safety priorities.

These findings support a tiered and context-sensitive approach to EMR use certification. A consistent set of patient safety objectives may be retained across institution types, but implementation guidance, evidence requirements, and support mechanisms may need to differ according to institutional readiness. For general hospitals and clinics, targeted support such as vendor participation, standardized implementation guidance, and financial incentives may be needed before use certification can be applied in a feasible and equitable manner.

The self-assessment checklist developed in this study should be positioned as a foundational component of EMR use certification rather than as a stand-alone substitute for all forms of use evaluations. The checklist provides a structured mechanism for documenting implementation status, governance, and improvement needs for patient safety-critical EMR functions. Scenario-based evaluations or direct workflow observations can complement the checklist by verifying how selected high-risk functions operate in real clinical contexts. Thus, the checklist can serve as a preparatory and screening tool that informs more targeted scenario-based evaluations.

### Strengths and Limitations

This study offers important insights into the development and content validation of a self-assessment checklist aligned with the Korean EMR Certification System; however, several limitations should be noted. First, the validation process primarily relied on the CVI. Although the CVI is a widely accepted method in instrument development and is supplemented by qualitative feedback from open-ended questions, it remains inherently subjective, as it is based on expert judgments. Although the modified κ was calculated to adjust each I-CVI for the probability of chance agreement, the modified κ is a chance-adjusted content-validity coefficient rather than a comprehensive measure of interrater reliability. Conventional interrater reliability metrics were not evaluated; therefore, expert agreement beyond the CVI-based acceptability assessment and chance-adjusted content-validity assessment could not be fully characterized. This approach may not fully capture the complexity of how the checklist performs in diverse, real-world health care settings. In addition, the number of valid ratings varied across item-dimension-institution–type combinations owing to missing ratings. Although missing ratings were excluded only from the specific calculations in which they occurred, differential nonresponse by institution type may have influenced some estimates, particularly for general hospital and clinic-level evaluations.

Second, the panel of experts, although experienced in health information systems and EMR certification, may not adequately represent the full spectrum of stakeholders involved in EMR implementation. Important perspectives from frontline clinical staff, EMR vendors, health care administrators, patient advocates, and policymakers were limited or absent, which may have influenced feedback regarding the checklist’s clarity, applicability, and usability. The expert panel was also unevenly distributed across stakeholder groups and health care institution types and was selected primarily on the basis of expertise in EMR certification and health information systems rather than representation across health care institution types. Some experts were not affiliated with health care institutions, and among those with health care institution affiliations, participants were concentrated in large tertiary or university-affiliated hospitals. Although the expert panel included a physician currently practicing in a primary care clinic, frontline representation from clinical settings remained limited. Accordingly, clinic-level findings should be interpreted cautiously as expert-assessed applicability rather than definitive evidence from a broad sample of frontline primary care clinicians or clinic managers.

Third, the study design required experts to provide numerous quantitative ratings across multiple dimensions and settings, as well as practical assessments and open-ended feedback. This may have imposed a high cognitive burden and contributed to survey fatigue, particularly influencing responses related to ease of completion.

Fourth, the validation was limited to content validity. Other important psychometric properties, such as construct validity, criterion-related validity, and reliability, were not evaluated. These aspects are essential for establishing the robustness, consistency, and generalizability of the checklist, especially if it is intended for use in formal evaluations or regulatory settings.

Finally, although the adaptation of the ONC checklist to the Korean context was carried out using a structured approach, it may not have fully captured certain localized clinical practices or regulatory nuances. Continuous refinement through pilot testing and real-world application in Korean health care settings is essential to ensure the checklist’s relevance and alignment with actual certification processes and clinical workflows.

Future research should empirically test the checklist across diverse health care environments, evaluate additional dimensions of validity and reliability, and examine how checklist use relates to implementation processes and patient safety-relevant outcomes. It will also be important to incorporate feedback from a broader range of end users, including frontline clinicians, administrators, EMR vendors, clinic managers, policymakers, and patients. These efforts may help determine whether the tool is practical, reliable, and useful for supporting EMR certification and patient safety–focused evaluation in routine clinical settings.

### Conclusions

This study developed and evaluated the content validity of a self-assessment checklist for EMR use certification focused on 10 patient safety–related certification items aligned with the Korean EMR Certification System. Expert evaluation demonstrated high overall content validity, supporting the relevance and appropriateness of the selected domains for assessing EMR use in clinical practice. However, perceived applicability and expert-assessed feasibility varied across tertiary referral hospitals, general hospitals, and clinics, suggesting that institutional context should be considered when applying the checklist. These results support the checklist as a foundational self-assessment tool for evaluating patient safety-critical aspects of EMR use. Further empirical testing is needed to examine its reliability, feasibility, and utility in real-world certification processes.

## Supplementary material

10.2196/95500 Multimedia Appendix 1 Self-assessment checklist for evaluating electronic medical record use certification items.

10.2196/95500 Multimedia Appendix 2 Complete item-level Content Validity Index, probability of chance agreement, and modified κ values by item, dimension, and health care institution type.

## Abbreviations

CVI: Content Validity Index
EHR: electronic health record
EMR: electronic medical record
I-CVI: item-level Content Validity Index
KHIS: Korea Health Information Service
ONC: US Office of the National Coordinator for Health Information Technology
RFID: radio frequency identification
S-CVI/Ave: scale-level Content Validity Index
WHO: World Health Organization
