# Supplementary material for: Development and Content Validation of a Self-Assessment Checklist for Electronic Medical Record Use Certification in Korea: Convergent Mixed Methods Study
Source: JMIR Med Inform. 2026 Nov 4;14:e95500. doi: 10.2196/95500 (PMC13734700; doi:10.2196/95500)
Supplement: Supplementary file 1 [file medinform-v14-e95500-s001.pdf]

Certification Item A003

Patient Identification: Display of Identifiers for Patients with Identical Names

Certification Item A003

Display of Identifiers for Patients with Identical Names

• Purpose of the Requirement

- The certification criteria for patient information management (A001–A003) require that an electronic medical record (EMR) system be equipped with essential functionalities to generate core demographic information at the initiation of care and to ensure accurate patient identification for the purpose of patient safety.

- The first information to be generated when initiating patient care is the patient registration number, which must be created upon entering the patient's demographic data. In particular, the registration number, patient name, date of birth, and sex constitute the minimum identification elements that must be displayed across all prescription and medical record screens throughout the care process to ensure patient safety.

- Furthermore, clinical information such as medical records must be managed consistently and uniformly through the patient registration number. To this end, medical records belonging to the same patient who has been issued multiple registration numbers should be consolidated for integrated retrieval as a single patient record.

- During clinical encounters, patients with identical names must be clearly distinguishable when selecting from the scheduled patient list, and the system must notify users when a duplicate name is present at the time of patient registration or retrieval. To enhance the accuracy of patient identification, systems may employ additional identification mechanisms such as barcode or RFID-enabled wristbands. Such mechanisms are also applied at the first step of the "Five Rights of Safe Medication" and are presented as an optional item under certification criterion A021.

- The patient information management certification criteria are mandatory requirements for obtaining EMR certification.

Certification Item A003

- Checklists

1. In order to alert or highlight when patients share the same or similar names, the system must include a function to display distinguishing identifiers.

	Fully implemented	Partially implemented	Not implemented
1-1. Does the system automatically generate and display distinguishing identifiers for patients with identical names?	☐	☐	☐
1-2. Does the distinguishing identifier include one or more of the following elements?<br>(1) Icon (2) Special character	☐	☐	☐
(3) Alphabetic marker (4) Color coding	☐	☐	☐
1-3. Does the system prevent users from manually altering the distinguishing identifiers assigned to patients with identical names?			

Improvement and Management Plan

-

-

-

-

[Reference for Completion]
Reference standard: When patients with identical or similar names are scheduled for the same outpatient visit, admitted to the same ward, or scheduled for surgery on the same date, the system must emphasize and clearly notify users.

Certification Item A003

- Checklists

2. The system must notify users when patients with identical names exist during patient search and allow users to select the correct patient directly.

	Fully implemented	Partially implemented	Not implemented
2-1. When identical names are found during a patient search, can the user review the list and directly select the intended patient?	☐	☐	☐
2-2. Can the system display patients with identical names across various types of patient lists (e.g., outpatient department lists, ward-based patient lists, surgical waiting lists)?	☐	☐	☐

Improvement and Management Plan

-

-

-

-

[Reference for Completion]
Reference standard: When a new patient is registered or searched, if another patient with the same name already exists, the system must present at least two additional distinguishing data elements (e.g., address, date of birth) in the search results. These additional identifiers should assist both the patient and the user in confirming the correct identity.

Certification Item A003

• Organizational Responsibility Checklist

- **[Responsible Department/Lead Personnel]**

When issues arise or system improvement requests are made regarding the display of distinguishing identifiers for patients with identical names, is there a designated department and responsible person who assume accountability and lead the process?

Established	Not established	In preparation
☐	☐	☐

- **[Operational Staff/Operational Department]**

When issues arise or system improvement requests are made regarding the display of distinguishing identifiers for patients with identical names, is there a designated department and staff member responsible for day-to-day operational tasks?

Established	Not established	In preparation
☐	☐	☐

- **[Collaboration/Stakeholder Departments]**

When issues arise or system improvement requests are made regarding the display of distinguishing identifiers for patients with identical names, are the other departments and staff members who must primarily collaborate with the responsible department clearly identified?

Established	Not established	In preparation
☐	☐	☐

- **[Coordinated Issue Resolution]**

When issues arise or system improvement requests are made regarding the display of distinguishing identifiers for patients with identical names, can the operational department and collaborating departments work together in an organized manner to resolve the problem?

Established	Not established	In preparation
☐	☐	☐

Certification Item A009

Recording, Editing, and Reviewing the Medication Allergy and Hypersensitivity List

Certification Item A009

Recording, Editing, and Reviewing the Medication Allergy and Hypersensitivity List

• Purpose of the Requirement

- The clinical information management certification criteria (A007–A009) require that an electronic medical record (EMR) system include functions for managing core patient lists, not only to cover information from a specific episode of care (e.g., inpatient stay or outpatient visit), but also to provide a comprehensive overview of the patient's current health problems in order to ensure patient-centered continuity of care. Core lists include the problem (diagnosis) list (A007), the medication list (A008), and the medication allergy list (A009). For continuity of care, these lists must integrate not only information generated within the healthcare institution but also relevant information received from external healthcare organizations. Furthermore, these core lists should remain available as essential information for patient safety, even in emergency situations where the EMR system is not fully operational.

- Medical records are structured information generated through establishing a care plan, providing services according to that plan, and subsequently measuring and documenting outcomes. They are fundamental for patient-centered continuity of care and communication among healthcare professionals, and therefore must be managed in a standardized format. This requirement also aligns with national health information policies aimed at establishing foundational infrastructure for the transition to digital healthcare.

- The EMR system must also include information necessary for external data sharing. Such information requirements are specified under major certification categories, including "2. Information Sharing Between Healthcare Institutions," "3. Patient Information Provision," and "5. Standards Management." Accordingly, the subcategory "1.3 Clinical Information Management" requires functionalities for the management of source data essential for external sharing.

- The clinical information management certification criteria distinguish between mandatory requirements—necessary for obtaining certification—and optional items, which serve as preparatory steps for transitioning to a digital healthcare environment.

Certification Item A009

- Checklists

1. In order to ensure safe medication administration, the EMR system must manage the patient's medication allergy list.

	Fully implemented	Partially implemented	Not implemented
1-1. When entering medication allergy information, does the system utilize the standardized medication code of the causative drug?	☐	☐	☐
1-2. If a medication allergy is recorded without a medication code, does the system notify users that no alert will be generated within the Clinical Decision Support (CDS) function?	☐	☐	☐
1-3. During the prescribing or medication administration process, does the system clearly indicate whether the patient has a medication allergy?	☐	☐	☐
1-4. Does the system include severity classification for medication allergies and hypersensitivity reactions (e.g., mild, moderate, severe)?	☐	☐	☐

Improvement and Management Plan

-

-

-

[Reference for Completion]

Coded allergy antigens and reaction information (or documentation of *No Known Allergies [NKA]*) must be entered and updated in the medical record before prescription entry. (221-05) / The system should enable clear entry of allergy information and facilitate easy documentation of "No Known Allergies." (221-04) / Allergy-related patient information should be automatically retrieved and displayed on the prescription entry screen. (312-01) / Critical patient information (e.g., diagnoses, current medications, allergies) must always be displayed and emphasized in the patient information view. (121-01)

Certification Item A009

- Checklists

2. For institutions with 300 or more beds, the EMR system must allow the use of standardized terminology (codes) when recording both the causative drug and the associated symptoms.

	Fully implemented	Partially implemented	Not implemented
2-1. Can the system record the name of the causative drug using standardized codes?<br>(Korea Drug Standard Code (KD),<br>Electronic Data Interchange Code (EDI),<br>World Health Organization Anatomical Therapeutic Chemical (WHO ATC))	☐	☐	☐
2-2. Can the system record symptoms using the 8th Korean Standard Classification of Diseases (KCD-8)?<br>(Additional coding systems such as SNOMED CT or MedDRA may also be utilized)	☐	☐	☐

Improvement and Management Plan

-

-

-

Certification Item A009

• Checklists

3. The medication allergy and hypersensitivity list must allow clear distinction and recording of the following statuses: *Present*, *Absent*, *Unknown, and Not Interviewed*.

	Fully implemented	Partially implemented	Not implemented
3-1. During patient interviews, if the patient reports having a medication allergy, is it recorded as *Present*? If the patient reports no allergy, is it recorded as *Absent*? If the patient is uncertain, is it recorded as *Unknown*?	☐	☐	☐
3-2. If no interview is conducted, is the status recorded as *Not Interviewed*?	☐	☐	☐

Improvement and Management Plan

-

-

-

[Reference for Completion]

•Ensure that ʺMissingʺ or ʺUnknownʺ categories are available to prevent inaccurate information entry. (220-02)

•The system must enable clear entry of allergy information and facilitate easy documentation of *No Known Allergies (NKA)*. (221-04)

Certification Item A009

- Checklists

4. To prevent adverse events, the registered medication allergy and hypersensitivity information must be shared with healthcare providers and clearly displayed on the patient's basic information screen using badges, icons, or similar visual markers.

	Fully implemented	Partially implemented	Not implemented
4-1. Is the registered medication allergy and hypersensitivity information displayed on the patient's basic information screen (e.g., with a badge, icon, or similar indicator)?	☐	☐	☐

Improvement and Management Plan

-

-

-

[Reference for Completion]

Critical patient information must always be displayed and emphasized on the patient information screen (e.g., diagnoses, current medications, allergies). (121-01)

Certification Item A009

• Organizational Responsibility Checklist

- **[Responsible Department/Lead Personnel]**

When issues arise or system improvement requests are made regarding the recording, editing, and reviewing of the medication allergy and hypersensitivity list, is there a designated department and responsible person who assume accountability and lead the process?

Established	Not established	In preparation
☐	☐	☐

- **[Operational Staff/Operational Department]**

When issues arise or system improvement requests are made regarding the recording, editing, and reviewing of the medication allergy and hypersensitivity list, is there a designated department and staff member responsible for day-to-day operational tasks?

Established	Not established	In preparation
☐	☐	☐

- **[Collaboration/Stakeholder Departments]**

When issues arise or system improvement requests are made regarding the recording, editing, and reviewing of the medication allergy and hypersensitivity list, are the other departments and staff members who must primarily collaborate with the responsible department clearly identified?

Established	Not established	In preparation
☐	☐	☐

- **[Coordinated Issue Resolution]**

When issues arise or system improvement requests are made regarding the recording, editing, and reviewing of the medication allergy and hypersensitivity list, can the operational department and collaborating departments work together in an organized manner to resolve the problem?

Established	Not established	In preparation
☐	☐	☐

Certification Item A017

Drug Interaction and Allergy Alert

Certification Item A017

Drug Interaction and Allergy Alert

• Purpose of the Requirement

- The clinical decision support (CDS) certification criteria (A017–A021) address functionalities related to medication safety. Among these, A017 requires that the EMR system provide checking functions to prevent adverse drug reactions by identifying (1) drug–drug interactions (between newly prescribed medications, or between prescribed and currently administered medications) and (2) drug–allergy interactions (between prescribed medications and causative agents of recorded allergies).

- Certification items A018–A021 further require implementation of the “Five Rights of Safe Medication” within the EMR system to prevent medication errors: right patient, right drug, right dose, right route, and right time. Specifically, certification item A021 requires functions to verify the right patient, right drug, and right time (with patient identification also addressed in A003). Meanwhile, A018, A019, and A020 address the management of right drug selection, right administration route, and right dosage, respectively, at the prescribing stage.

- The development of CDS functionalities can be facilitated through the use of standardized medical data. A representative example is the utilization of DICOM standards in radiology to support image interpretation in computer-aided diagnosis (CADx) solutions. Similarly, CDS algorithms developed based on standardized clinical information and integrated into practice can provide patients with safe, high-quality, evidence-based care, thereby contributing to the establishment of a digital healthcare innovation ecosystem. Overall, CDS functionalities contribute to critical healthcare objectives, including improving patient safety, healthcare quality, equity, and accessibility.

- The CDS certification criteria distinguish between mandatory and optional requirements depending on healthcare institution type and certification category. Institutions preparing for digital transformation may adopt the optional functions as part of their readiness efforts.

Certification Item A017

- Checklists

1. Drug–drug interaction alerts must be based on the patient's medication list (A008) and executed at the time of prescribing new medications.

	Fully implemented	Partially implemented	Not implemented
1-1. When the patient is already taking another medication that has a clinically significant potential interaction with the newly prescribed drug, does the system generate an alert for the user according to predefined rules? (Including contrast agents used for imaging studies)	☐	☐	☐
1-2. Are drug–allergy checks and alerts based on the patient's medication allergy list (A009), with rules applied to identify same ingredients, ingredient classes, or cross-reactive groups, and are such checks executed at the time of prescribing new medications? (May also include adverse drug reaction checks)	☐	☐	☐

Improvement and Management Plan

-

-

-

[Reference for Completion]
If the patient is taking another drug with a clinically significant potential interaction with the newly prescribed medication, the system must alert the prescriber. (Including contrast agents used for imaging studies) (421-02)
Alerts must be provided for contraindications due to allergy history and cross-allergies. (421-02)

Certification Item A017

- Checklists

2. When a user proceeds with prescribing despite a drug–drug interaction alert, the system must require documentation of the prescribing rationale.

2-1. If a prescription is entered despite the alert, does the system automatically display a screen prompting the prescriber to enter the rationale for overriding the alert?

Fully implemented	Partially implemented	Not implemented
☐	☐	☐

Improvement and Management Plan

-

-

-

[Reference for Completion]
For alerts that are frequently overridden, the system should provide an editing function within the medical record that includes links to update allergy information and the medication list. (522-03)

Certification Item A017

- Checklists

3. Excessive alerts may cause alert fatigue among clinicians and potentially compromise patient safety. Therefore, drug–drug interaction and drug–allergy alert rules (e.g., severity levels, requirement for rationale entry) should be configurable by the healthcare institution according to clinical importance and priority, and adjustable by user role. *(This item may be reviewed optionally; even if reviewed, the results will not be included in certification requirements.)*

	Fully implemented	Partially implemented	Not implemented
3-1. Does the system provide functionality that allows healthcare institutions to configure drug–drug interaction and drug–allergy alert rules (e.g., severity levels, rationale entry) based on clinical importance and priority, and to adjust them according to user type?	☐	☐	☐
3-2. Does the system include role-based authorization functions that permit only authorized users to configure alert rules?	☐	☐	☐

Improvement and Management Plan

-

-

-

[Reference for Completion]

Alert rules must allow prioritization so that safety alerts can be ranked according to clinical importance. (411-01)

Certification Item A017

- ## Organizational Responsibility Checklist

- **[Responsible Department/Lead Personnel]**

When issues arise or system improvement requests are made regarding drug interaction and allergy alert functions, is there a designated department and responsible person who assume accountability and lead the process?

Established	Not established	In preparation
☐	☐	☐

- **[Operational Staff/Operational Department]**

When issues arise or system improvement requests are made regarding drug interaction and allergy alert functions, is there a designated department and staff member responsible for day-to-day operational tasks?

Established	Not established	In preparation
☐	☐	☐

- **[Collaboration/Stakeholder Departments]**

When issues arise or system improvement requests are made regarding drug interaction and allergy alert functions, are the other departments and staff members who must primarily collaborate with the responsible department clearly identified?

Established	Not established	In preparation
☐	☐	☐

- **[Coordinated Issue Resolution]**

When issues arise or system improvement requests are made regarding drug interaction and allergy alert functions, can the operational department and collaborating departments work together in an organized manner to resolve the problem?

Established	Not established	In preparation
☐	☐	☐

Certification Item A018

Medication Entry Error Prevention

Certification Item A018

Medication Entry Error Prevention

• Purpose of the Requirement

- The clinical decision support (CDS) certification criteria (A017–A021) encompass functionalities related to medication safety. Among these, A017 requires drug–drug and drug–allergy interaction checks to prevent adverse drug reactions. Certification items A018 through A021 address the prevention of medication errors by implementing the "Five Rights of Safe Medication" within the EMR system: right patient, right drug, right dose, right route, and right time. Specifically, A021 requires functionalities to verify the right patient, right drug, and right time (with patient identification also addressed in A003). At the prescribing stage, A018, A019, and A020 respectively require system functionalities to manage the right drug selection, right administration route, and right dosage.

- The development of CDS functionalities can be facilitated by the use of standardized medical data. A representative example is the use of the DICOM imaging standard in radiology to support interpretation through computer-aided diagnosis (CADx) solutions. Likewise, CDS algorithms developed with standardized clinical information and applied in practice can provide patients with safe, high-quality, evidence-based care, thereby fostering a digital healthcare innovation ecosystem. Overall, CDS functionalities contribute to critical healthcare goals, including the enhancement of patient safety, healthcare quality, equity, and accessibility.

- The CDS certification criteria distinguish between mandatory and optional requirements depending on healthcare institution type and certification category. Institutions preparing for digital transformation may adopt the optional functions as part of their readiness efforts.

Certification Item A018

- Checklists

1. Certification criterion A018 requires the EMR system to include error-prevention functions that ensure the correct drug is prescribed, in alignment with the “Five Rights of Safe Medication” as stipulated under Article 58 of the Medical Service Act (Healthcare Institution Accreditation). These rights are: the right patient, the right drug, the right time, the right dose, and the right route.

1-1. During medication prescribing, can drug names be managed and configured in the drug master in order to prevent entry errors?	Fully implemented	Partially implemented	Not implemented
	☐	☐	☐

Improvement and Management Plan

-

-

-

Certification Item A018

- Checklists

2. To prevent entry errors related to similar drug names, the EMR system must implement mechanisms to distinguish and clearly display drug names. These methods include:
(1) Differentiation of look-alike/sound-alike drug names (e.g., Tall Man Lettering, bilingual display in English/Korean),
(2) Differentiated display for drugs with multiple strengths (e.g., color or shape markers),
(3) Differentiated display for drugs with multiple formulations (e.g., formulation name in Korean), and
(4) Distinct display for high-alert or high-risk medications.

	Fully implemented	Partially implemented	Not implemented
2-1. For look-alike/sound-alike drug names, does the EMR system implement at least one of the following methods?<br>Tall Man Lettering, Identifier markers, Color-coded drug names, Shape markers displayed next to the drug name, Bilingual display(English/Korean)	☐	☐	☐
2-2. For drugs with multiple strengths or formulations, does the EMR system implement at least one of the following methods?<br>-Displaying all strengths or formulations in search results<br>-Adding symbols or strength labels to drug names<br>-Displaying formulation names in Korean<br>-Color-coding drug names	☐	☐	☐
2-3. Are high-alert or high-risk medications clearly distinguished and displayed separately from general medications?	☐	☐	☐

Improvement and Management Plan

Certification Item A018

- Checklists

3. When configuring the detailed settings for differentiated display of drug names, the same management method must not be duplicated across categories.

- 3-1. In cases where a drug is both a *look-alike/sound-alike medication* and a *multi-strength medication*, if “color-coding” is used as the management method for one category, is a different method (excluding color-coding) applied for the other category?

Fully implemented	Partially implemented	Not implemented
☐	☐	☐

Improvement and Management Plan

-

-

-

Certification Item A018

- Checklists

4. To strengthen medication error prevention, it is recommended that the functions required under certification criterion A018 be implemented not only at the prescribing stage but also during the dispensing/review and administration stages.

- 4-1. Has the system implemented the functions of certification criterion A018 for medication error prevention across all stages of the medication process—prescribing, dispensing/review, and administration?

Fully implemented	Partially implemented	Not implemented
☐	☐	☐

Improvement and Management Plan

-

-

-

Certification Item A018

- ## Organizational Responsibility Checklist

- **[Responsible Department/Lead Personnel]**

When issues arise or system improvement requests are made regarding medication entry error prevention, is there a designated department and responsible person who assume accountability and lead the process?

Established	Not established	In preparation
☐	☐	☐

- **[Operational Staff/Operational Department]**

When issues arise or system improvement requests are made regarding medication entry error prevention, is there a designated department and staff member responsible for day-to-day operational tasks?

Established	Not established	In preparation
☐	☐	☐

- **[Collaboration/Stakeholder Departments]**

When issues arise or system improvement requests are made regarding medication entry error prevention, are the other departments and staff members who must primarily collaborate with the responsible department clearly identified?

Established	Not established	In preparation
☐	☐	☐

- **[Coordinated Issue Resolution]**

When issues arise or system improvement requests are made regarding medication entry error prevention, can the operational department and collaborating departments work together in an organized manner to resolve the problem?

Established	Not established	In preparation
☐	☐	☐

Certification Item A019

Inappropriate Route of Administration Management

Certification Item A019

Inappropriate Route of Administration Management

• Purpose of the Requirement

- The clinical decision support (CDS) certification criteria (A017–A021) encompass functionalities related to medication safety. Among these, A017 requires drug–drug and drug–allergy interaction checks to prevent adverse drug reactions. Certification items A018 through A021 address the prevention of medication errors by implementing the “Five Rights of Safe Medication” within the EMR system: right patient, right drug, right dose, right route, and right time. Specifically, A021 requires functionalities to verify the right patient, right drug, and right time (with patient identification also addressed in A003). At the prescribing stage, A018, A019, and A020 respectively require system functionalities to manage the right drug selection, right administration route, and right dosage.

- The development of CDS functionalities can be facilitated by the use of standardized medical data. A representative example is the use of the DICOM imaging standard in radiology to support interpretation through computer-aided diagnosis (CADx) solutions. Likewise, CDS algorithms developed with standardized clinical information and applied in practice can provide patients with safe, high-quality, evidence-based care, thereby fostering a digital healthcare innovation ecosystem. Overall, CDS functionalities contribute to critical healthcare goals, including the enhancement of patient safety, healthcare quality, equity, and accessibility.

- The CDS certification criteria distinguish between mandatory and optional requirements depending on healthcare institution type and certification category. Institutions preparing for digital transformation may adopt the optional functions as part of their readiness efforts.

Certification Item A019

- Checklists

1. The EMR system must provide functionalities to suggest appropriate default routes of administration for medications at the time of prescribing.

	Fully implemented	Partially implemented	Not implemented
1-1. Is the default route of administration for each medication configured and managed within the drug master?	☐	☐	☐
1-2. Can the system configure and manage mandatory or contraindicated routes of administration for each medication according to institutional policy?	☐	☐	☐

Improvement and Management Plan

-

-

-

[Reference for Completion]
Default values for drug, dose, frequency, and route should only be used when they are always correct. (323-02)
If an inappropriate route of administration is prescribed for a given medication, the system must display a warning. (422-11)

Certification Item A019

- Checklists

2. The EMR system must ensure that only clinically appropriate and recommended routes of administration can be selected for each medication in order to prevent prescribing errors. If an inappropriate route is selected, the system must provide a check and generate an alert.

	Fully implemented	Partially implemented	Not implemented
2-1. Does the system restrict selectable routes of administration to clinically appropriate or recommended routes for each medication, or generate an alert when an inappropriate route is selected?	☐	☐	☐
2-2. To prevent alert fatigue, which can compromise patient safety, does the system allow healthcare institutions to configure alert rules (e.g., severity levels, prioritization) so that clinically important and high-priority alerts are emphasized, while unnecessary alerts are limited?	☐	☐	☐

Improvement and Management Plan

-

-

-

[Reference for Completion]

Default values for drug, dose, frequency, and route should only be used when they are always correct. (323-02)

The number and type of alerts should be limited to those clinically essential for safe and effective patient care. (411-02)

Critical alerts should be designed to be difficult to override. (411-03)

Alerts essential to patient safety must not be disabled. (411-04)

Certification Item A019

- ## Organizational Responsibility Checklist

- **[Responsible Department/Lead Personnel]**

When issues arise or system improvement requests are made regarding inappropriate route of administration management, is there a designated department and responsible person who assume accountability and lead the process?

Established	Not established	In preparation
☐	☐	☐

- **[Operational Staff/Operational Department]**

When issues arise or system improvement requests are made regarding inappropriate route of administration management, is there a designated department and staff member responsible for day-to-day operational tasks?

☐	☐	☐

- **[Collaboration/Stakeholder Departments]**

When issues arise or system improvement requests are made regarding inappropriate route of administration management, are the other departments and staff members who must primarily collaborate with the responsible department clearly identified?

☐	☐	☐

- **[Coordinated Issue Resolution]**

When issues arise or system improvement requests are made regarding inappropriate route of administration management, can the operational department and collaborating departments work together in an organized manner to resolve the problem?

☐	☐	☐

Certification Item A020

Safe Prescription of Medication Dosages

Certification Item A020

Safe Prescription of Medication Dosages

• Purpose of the Requirement

- The clinical decision support (CDS) certification criteria (A017–A021) encompass functionalities related to medication safety. Among these, A017 requires drug–drug and drug–allergy interaction checks to prevent adverse drug reactions. Certification items A018 through A021 address the prevention of medication errors by implementing the "Five Rights of Safe Medication" within the EMR system: right patient, right drug, right dose, right route, and right time. Specifically, A021 requires functionalities to verify the right patient, right drug, and right time (with patient identification also addressed in A003). At the prescribing stage, A018, A019, and A020 respectively require system functionalities to manage the right drug selection, right administration route, and right dosage.

- The development of CDS functionalities can be facilitated by the use of standardized medical data. A representative example is the use of the DICOM imaging standard in radiology to support interpretation through computer-aided diagnosis (CADx) solutions. Likewise, CDS algorithms developed with standardized clinical information and applied in practice can provide patients with safe, high-quality, evidence-based care, thereby fostering a digital healthcare innovation ecosystem. Overall, CDS functionalities contribute to critical healthcare goals, including the enhancement of patient safety, healthcare quality, equity, and accessibility.

- The CDS certification criteria distinguish between mandatory and optional requirements depending on healthcare institution type and certification category. Institutions preparing for digital transformation may adopt the optional functions as part of their readiness efforts.

Certification Item A020

- Checklists

1. When prescribing medications, the EMR system must support the calculation of recommended doses for each patient based on one or more anthropometric or clinical parameters.

	Fully implemented	Partially implemented	Not implemented
1-1. When prescribing a medication, can the system calculate patient-specific recommended doses based on one or more of the following? (Body weight, Age, Body surface area (BSA), Chemotherapy protocols, Diagnosis, Renal function)	☐	☐	☐
1-2. Does the system provide a separate dosage calculator within the EMR, or a function within the prescribing interface that automatically calculates the appropriate medication dose?	☐	☐	☐
1-3. When patient weight data already exist in the EMR, is the most recent measurement prior to the prescription date automatically retrieved for dose calculation?	☐	☐	☐
1-4. For pediatric prescriptions, does the system support dose calculation using one of the following sources? (Pediatric dosage by body weight, Pediatric dosage by BSA, Pediatric-specific fixed dosage)	☐	☐	☐
1-5. For chemotherapy prescriptions, does the system support recommended dose calculations using one of the following? (BSA, Ideal body weight (IBW), Adjusted body weight (ABW))	☐	☐	☐

Certification Item A020

- Checklists

Improvement and Management Plan

-

-

-

-

-

Certification Item A020

- Checklists

2. To ensure safe medication administration, the system must be able to link the calculated medication dose directly with the prescribing process.

2-1. Can the system integrate the calculated safe medication dose with the prescription entry process?

Fully implemented	Partially implemented	Not implemented
☐	☐	☐

Improvement and Management Plan

-

-

-

[Reference for Completion]
Default values for drug, dose, frequency, and route should only be used when they are always correct. (323-02)
The number and type of alerts should be limited to those clinically essential for safe and effective patient care. (411-02)
Critical alerts should be designed to be difficult to override. (411-03)
Alerts essential to patient safety must not be disabled. (411-04)

Certification Item A020

- Checklists

3. When prescribing medications, the system must provide functionalities that allow users to review or access dosage range information (e.g., maximum single dose or maximum daily dose) to ensure safe administration.

3-1. When prescribing a medication, does the system provide functionalities that allow users to review or access dosage range information (e.g., maximum single dose or maximum daily dose)?

Fully implemented	Partially implemented	Not implemented
☐	☐	☐

Improvement and Management Plan

-

-

-

Certification Item A020

- ## Organizational Responsibility Checklist

- **[Responsible Department/Lead Personnel]**

When issues arise or system improvement requests are made regarding the safe prescription of medication dosages, is there a designated department and responsible person who assume accountability and lead the process?

Established	Not established	In preparation
☐	☐	☐

- **[Operational Staff/Operational Department]**

When issues arise or system improvement requests are made regarding the safe prescription of medication dosages, is there a designated department and staff member responsible for day-to-day operational tasks?

Established	Not established	In preparation
☐	☐	☐

- **[Collaboration/Stakeholder Departments]**

When issues arise or system improvement requests are made regarding the safe prescription of medication dosages, are the other departments and staff members who must primarily collaborate with the responsible department clearly identified?

Established	Not established	In preparation
☐	☐	☐

- **[Coordinated Issue Resolution]**

When issues arise or system improvement requests are made regarding the safe prescription of medication dosages, can the operational department and collaborating departments work together in an organized manner to resolve the problem?

Established	Not established	In preparation
☐	☐	☐

Certification Item A021

Barcode or RFID-Based Safe Medication Administration

Certification Item A021

Barcode or RFID-Based Safe Medication Administration

• Purpose of the Requirement

- The clinical decision support (CDS) certification criteria (A017–A021) encompass functionalities related to medication safety. Among these, A017 requires drug–drug and drug–allergy interaction checks to prevent adverse drug reactions. Certification items A018 through A021 address the prevention of medication errors by implementing the "Five Rights of Safe Medication" within the EMR system: right patient, right drug, right dose, right route, and right time. Specifically, A021 requires functionalities to verify the right patient, right drug, and right time (with patient identification also addressed in A003).

- The development of CDS functionalities can be facilitated by the use of standardized medical data. A representative example is the use of the DICOM imaging standard in radiology to support interpretation through computer-aided diagnosis (CADx) solutions. Likewise, CDS algorithms developed with standardized clinical information and applied in practice can provide patients with safe, high-quality, evidence-based care, thereby fostering a digital healthcare innovation ecosystem. Overall, CDS functionalities contribute to critical healthcare goals, including the enhancement of patient safety, healthcare quality, equity, and accessibility.

- The CDS certification criteria distinguish between mandatory and optional requirements depending on healthcare institution type and certification category. Institutions preparing for digital transformation may adopt the optional functions as part of their readiness efforts.

Certification Item A021

- Checklists

1. The EMR system must include functionalities that utilize electronic identification devices (barcode or RFID) to verify the principles of safe medication administration and to generate administration records.

	Fully implemented	Partially implemented	Not implemented
1-1. Does the system include functionalities that utilize electronic identification devices (barcode or RFID) to verify the principles of safe medication administration and to generate administration records?	☐	☐	☐
1-2. Do electronic identification devices (barcode or RFID) implement functionalities that verify at least the following three of the "Five Rights of Safe Medication" to prevent medication errors?<br>(Right patient, Right drug, Right time)	☐	☐	☐

Improvement and Management Plan

-

-

-

Certification Item A021

- Checklists

2. When medication administration information does not match patient information, prescription details, or dispensing records, the system must provide alerts notifying users of the discrepancy.

	Fully implemented	Partially implemented	Not implemented
2-1. When medication administration information does not match patient information, prescription information, or dispensing information, does the system provide alerts to notify users of the discrepancy?	☐	☐	☐
2-2. Do the discrepancy alerts include the following information? (Patient information, Medication information, Dosage, Route of administration, Scheduled administration time, Prescription status)	☐	☐	☐

Improvement and Management Plan

-

-

-

Certification Item A021

- Checklists

3. The EMR system must include functionalities to record and retrieve medication administration activities.

	Fully implemented	Partially implemented	Not implemented
3-1. Are records of medication administration, in accordance with the principles of safe medication, automatically generated?	☐	☐	☐
3-2. Does the system provide functionalities to retrieve necessary information at each stage of the medication process—prescription, dispensing, and administration?	☐	☐	☐
3-3. After administration, can the system display the performer’s name, execution status, and date/time of administration?	☐	☐	☐

Improvement and Management Plan

-

-

-

Certification Item A021

- Checklists

4. The EMR system must include functionalities to document reasons when a medication is not administered.

	Fully implemented	Partially implemented	Not implemented
4-1. Before completion of the medication administration process, does the system automatically display an input screen to record reasons for non-administration?	☐	☐	☐
4-2. When entering a reason for non-administration, does the system allow users to review the processing status of the medication and include a function to return medications that were not administered?	☐	☐	☐
4-3. When a medication is not administered, does the system provide standardized reasons that include at least the following: IV route not available, on hold, discontinued, NPO (nothing by mouth), discharged, refusal, or drug unavailable?	☐	☐	☐

Improvement and Management Plan

-

-

-

Certification Item A021

• Organizational Responsibility Checklist

- **[Responsible Department/Lead Personnel]**

When issues arise or system improvement requests are made regarding barcode or RFID-based safe medication administration, is there a designated department and responsible person who assume accountability and lead the process?

Established	Not established	In preparation
☐	☐	☐

- **[Operational Staff/Operational Department]**

When issues arise or system improvement requests are made regarding barcode or RFID-based safe medication administration, is there a designated department and staff member responsible for day-to-day operational tasks?

☐	☐	☐

- **[Collaboration/Stakeholder Departments]**

When issues arise or system improvement requests are made regarding barcode or RFID-based safe medication administration, are the other departments and staff members who must primarily collaborate with the responsible department clearly identified?

☐	☐	☐

- **[Coordinated Issue Resolution]**

When issues arise or system improvement requests are made regarding barcode or RFID-based safe medication administration, can the operational department and collaborating departments work together in an organized manner to resolve the problem?

☐	☐	☐

Certification Item A022

Provision of Medication Information

Certification Item A022

Provision of Medication Information

• Purpose of the Requirement

- The clinical decision support (CDS) certification criteria A022–A023 require the EMR system to provide essential information during the medication use process. Specifically, A022 requires functionalities to provide drug information at the prescribing, dispensing, and administration stages, while A023 requires functionalities to provide tailored educational materials to enhance patient adherence to treatment. These functions are designed to ensure that necessary clinical information is delivered efficiently throughout the care process.

- The development of CDS functionalities can be facilitated by the use of standardized medical data. A representative example is the use of the DICOM imaging standard in radiology to support interpretation through computer-aided diagnosis (CADx) solutions. Likewise, CDS algorithms developed with standardized clinical information and applied in practice can provide patients with safe, high-quality, evidence-based care, thereby fostering a digital healthcare innovation ecosystem. Overall, CDS functionalities contribute to critical healthcare goals, including the enhancement of patient safety, healthcare quality, equity, and accessibility.

- The CDS certification criteria distinguish between mandatory and optional requirements depending on healthcare institution type and certification category. Institutions preparing for digital transformation may adopt the optional functions as part of their readiness efforts.

Certification Item A022

- Checklists

1. Medication information refers to data provided based on the Ministry of Food and Drug Safety (MFDS) product approval specifications. This may also include information from third-party providers of drug information, in addition to MFDS resources.

	Fully implemented	Partially implemented	Not implemented
1-1. At the stages of prescribing, dispensing, and administration, can users retrieve drug information?	☐	☐	☐
1-2. Although the specific information fields may vary by healthcare institution, does the system include at least the following elements in its drug information?<br>(Active ingredients and their amounts,<br>Physical characteristics (form, appearance),<br>Indications and therapeutic effects,<br>Dosage and administration,<br>Precautions for use,<br>Packaging unit, Storage method and expiration (shelf-life),<br>Manufacturer information)	☐	☐	☐

Improvement and Management Plan

-

-

-

[Reference for Completion]
Provide users with the evidence underlying default values. (323-03)

Certification Item A022

• Organizational Responsibility Checklist

- **[Responsible Department/Lead Personnel]**

When issues arise or system improvement requests are made regarding the provision of medication information, is there a designated department and responsible person who assume accountability and lead the process?

Established	Not established	In preparation
☐	☐	☐

- **[Operational Staff/Operational Department]**

When issues arise or system improvement requests are made regarding the provision of medication information, is there a designated department and staff member responsible for day-to-day operational tasks?

☐	☐	☐

- **[Collaboration/Stakeholder Departments]**

When issues arise or system improvement requests are made regarding the provision of medication information, are the other departments and staff members who must primarily collaborate with the responsible department clearly identified?

☐	☐	☐

- **[Coordinated Issue Resolution]**

When issues arise or system improvement requests are made regarding the provision of medication information, can the operational department and collaborating departments work together in an organized manner to resolve the problem?

☐	☐	☐

Certification Item A024

Critical Test Result Notification

Certification Item A024

Critical Test Result Notification

• Purpose of the Requirement

- The clinical decision support (CDS) certification criteria A024–A025 require the EMR system to support essential functions for patient safety. Specifically, A024 requires that critical test results be promptly and accurately communicated to the responsible healthcare professional, while A025 requires the implementation of antibiotic prescription management in accordance with the “Antimicrobial Stewardship Program” established by the Korea Disease Control and Prevention Agency and other authorities, in order to reduce antimicrobial resistance.

- The development of CDS functionalities can be facilitated by the use of standardized medical data. A representative example is the use of the DICOM imaging standard in radiology to support interpretation through computer-aided diagnosis (CADx) solutions. Likewise, CDS algorithms developed with standardized clinical information and applied in practice can provide patients with safe, high-quality, evidence-based care, thereby fostering a digital healthcare innovation ecosystem. Overall, CDS functionalities contribute to critical healthcare goals, including the enhancement of patient safety, healthcare quality, equity, and accessibility.

- The CDS certification criteria distinguish between mandatory and optional requirements depending on healthcare institution type and certification category. Institutions preparing for digital transformation may adopt the optional functions as part of their readiness efforts.

Certification Item A024

- Checklists

1. Clinically significant abnormal test results must generate automated alerts, and confirmation of receipt must be ensured.

	Fully implemented	Partially implemented	Not implemented
1-1. Does the system include functionalities to urgently notify healthcare professionals of critical abnormal test results?	☐	☐	☐
1-2. Does the system ensure that critical alerts are acknowledged by providing feedback mechanisms for confirmation of test result receipt?	☐	☐	☐

Improvement and Management Plan

-

-

-

[Reference for Completion]
Automated test result alerts must be limited to clinically relevant findings. (412-03)
Provide alerts for serious abnormal test results. (431-01)
Urgent clinical information must be delivered to clinicians in a timely manner, with documentation of delivery recorded in the medical record. (431-02)

Certification Item A024

• Organizational Responsibility Checklist

- **[Responsible Department/Lead Personnel]**

When issues arise or system improvement requests are made regarding critical test result notifications, is there a designated department and responsible person who assume accountability and lead the process?

Established	Not established	In preparation
☐	☐	☐

- **[Operational Staff/Operational Department]**

When issues arise or system improvement requests are made regarding critical test result notifications, is there a designated department and staff member responsible for day-to-day operational tasks?

☐	☐	☐

- **[Collaboration/Stakeholder Departments]**

When issues arise or system improvement requests are made regarding critical test result notifications, are the other departments and staff members who must primarily collaborate with the responsible department clearly identified?

☐	☐	☐

- **[Coordinated Issue Resolution]**

When issues arise or system improvement requests are made regarding critical test result notifications, can the operational department and collaborating departments work together in an organized manner to resolve the problem?

☐	☐	☐

Certification Item A025

Antibiotic Prescription Management

Certification Item A025

Antibiotic Prescription Management

• Purpose of the Requirement

- The clinical decision support (CDS) certification criteria A024–A025 require the EMR system to support essential functions for patient safety. Specifically, A024 requires that critical test results be promptly and accurately communicated to the responsible healthcare professional, while A025 requires the implementation of antibiotic prescription management in accordance with the "Antimicrobial Stewardship Program" established by the Korea Disease Control and Prevention Agency and other authorities, in order to reduce antimicrobial resistance.

- The development of CDS functionalities can be facilitated by the use of standardized medical data. A representative example is the use of the DICOM imaging standard in radiology to support interpretation through computer-aided diagnosis (CADx) solutions. Likewise, CDS algorithms developed with standardized clinical information and applied in practice can provide patients with safe, high-quality, evidence-based care, thereby fostering a digital healthcare innovation ecosystem. Overall, CDS functionalities contribute to critical healthcare goals, including the enhancement of patient safety, healthcare quality, equity, and accessibility.

- The CDS certification criteria distinguish between mandatory and optional requirements depending on healthcare institution type and certification category. Institutions preparing for digital transformation may adopt the optional functions as part of their readiness efforts.

Certification Item A025

- Checklists

1. When prescribing antibiotics, the EMR system must provide automated notifications to ensure appropriate use.

1-1. When prescribing antibiotics or related medications, does the system provide an alert indicating that approval (or review) is required, so that prescriptions can only be issued to patients when they have been authorized for use in relevant specialties (e.g., infectious diseases, or pediatric infectious diseases for pediatric patients)?	Fully implemented	Partially implemented	Not implemented
	☐	☐	☐

Improvement and Management Plan

-

-

-

Certification Item A025

• Organizational Responsibility Checklist

- **[Responsible Department/Lead Personnel]**

When issues arise or system improvement requests are made regarding antibiotic prescription management, is there a designated department and responsible person who assume accountability and lead the process?

Established	Not established	In preparation
☐	☐	☐

- **[Operational Staff/Operational Department]**

When issues arise or system improvement requests are made regarding antibiotic prescription management, is there a designated department and staff member responsible for day-to-day operational tasks?

☐	☐	☐

- **[Collaboration/Stakeholder Departments]**

When issues arise or system improvement requests are made regarding antibiotic prescription management, are the other departments and staff members who must primarily collaborate with the responsible department clearly identified?

☐	☐	☐

- **[Coordinated Issue Resolution]**

When issues arise or system improvement requests are made regarding antibiotic prescription management, can the operational department and collaborating departments work together in an organized manner to resolve the problem?

☐	☐	☐
