# Supplementary material for: Development and Content Validation of a Self-Assessment Checklist for Electronic Medical Record Use Certification in Korea: Convergent Mixed Methods Study
Source: JMIR Med Inform. 2026 Nov 4;14:e95500. doi: 10.2196/95500 (PMC13734700; doi:10.2196/95500)
Supplement: Supplementary file 2 [file medinform-v14-e95500-s002.pdf]

Appendix 2. Item-Level I-CVI, Probability of Chance Agreement, and Modified Kappa by Evaluation Dimension and Institution Type.

			Relevance			*Usefulness*			*Clarity*			*Importance*		
Item	*Abbreviation*	*Institution type*	*I-CVI*	*Pc*	*κ**	*I-CVI*	*Pc*	*κ**	*I-CVI*	*Pc*	*κ**	*I-CVI*	*Pc*	*κ**
A003	PI	Tertiary	0.958	0.000001	0.958	0.958	0.000001	0.958	0.917	0.000016	0.917	0.917	0.000016	0.917
A003	PI	General	0.952	0.00001	0.952	0.952	0.00001	0.952	0.952	0.00001	0.952	0.952	0.00001	0.952
A003	PI	Clinics	0.952	0.00001	0.952	0.952	0.00001	0.952	0.905	0.0001	0.905	0.952	0.00001	0.952
A009	DAH	Tertiary	1	0	1	1	0	1	1	0	1	1	0	1
A009	DAH	General	1	0	1	1	0	1	1	0	1	1	0	1
A009	DAH	Clinics	1	0	1	1	0	1	0.952	0.00001	0.952	0.952	0.00001	0.952
A017	DIA	Tertiary	1	0	1	1	0	1	1	0	1	1	0	1
A017	DIA	General	1	0	1	1	0	1	1	0	1	1	0	1
A017	DIA	Clinics	0.905	0.0001	0.905	0.762	0.009703	0.76	0.905	0.0001	0.905	0.905	0.0001	0.905
A018	MEP	Tertiary	1	0	1	1	0	1	1	0	1	1	0	1
A018	MEP	General	1	0	1	1	0	1	1	0	1	1	0	1
A018	MEP	Clinics	1	0	1	0.81	0.002854	0.809	0.905	0.0001	0.905	0.857	0.000634	0.857
A019	IRAM	Tertiary	0.958	0.000001	0.958	0.958	0.000001	0.958	1	0	1	0.958	0.000001	0.958
A019	IRAM	General	0.952	0.00001	0.952	0.952	0.00001	0.952	1	0	1	0.952	0.00001	0.952
A019	IRAM	Clinics	0.952	0.00001	0.952	0.714	0.025875	0.707	0.857	0.000634	0.857	0.857	0.000634	0.857
A020	SPMD	Tertiary	1	0	1	0.958	0.000001	0.958	1	0	1	0.958	0.000001	0.958
A020	SPMD	General	1	0	1	1	0	1	1	0	1	1	0	1
A020	SPMD	Clinics	0.952	0.00001	0.952	0.81	0.002854	0.809	0.905	0.0001	0.905	0.905	0.0001	0.905
A021	BRMA	Tertiary	1	0	1	0.917	0.000016	0.917	0.917	0.000016	0.917	0.958	0.000001	0.958
A021	BRMA	General	0.905	0.0001	0.905	0.81	0.002854	0.809	0.905	0.0001	0.905	0.857	0.000634	0.857
A021	BRMA	Clinics	0.667	0.055447	0.647	0.476	0.168188	0.37	0.619	0.097032	0.578	0.667	0.055447	0.647
A022	MIP	Tertiary	1	0	1	0.958	0.000001	0.958	0.958	0.000001	0.958	1	0	1
A022	MIP	General	1	0	1	0.952	0.00001	0.952	0.952	0.00001	0.952	0.952	0.00001	0.952
A022	MIP	Clinics	0.857	0.000634	0.857	0.81	0.002854	0.809	0.857	0.000634	0.857	0.81	0.002854	0.809
A024	CTRN	Tertiary	1	0	1	1	0	1	1	0	1	1	0	1
A024	CTRN	General	1	0	1	1	0	1	1	0	1	1	0	1
A024	CTRN	Clinics	0.952	0.00001	0.952	0.762	0.009703	0.76	0.81	0.002854	0.809	0.81	0.002854	0.809
A025	APM	Tertiary	0.917	0.000016	0.917	0.917	0.000016	0.917	0.917	0.000016	0.917	0.958	0.000001	0.958
A025	APM	General	0.857	0.000634	0.857	0.905	0.0001	0.905	0.857	0.000634	0.857	0.905	0.0001	0.905
A025	APM	Clinics	0.524	0.168188	0.428	0.476	0.168188	0.37	0.667	0.055447	0.647	0.667	0.055447	0.647

Note: I-CVI=item-level content validity index; Pc=probability of chance agreement; K=modified kappa.
I-CVI=A/N, where A is the number of ratings of 3 or 4 and N is the number of valid ratings.
Pc=C(N,A)(0.5)^N; K*=(I-CVI-Pc)/(1-Pc).
